# Development of an Oral Compound Pickering Emulsion Composed of Nanocrystals of Poorly Soluble Ingredient and Volatile Oils from Traditional Chinese Medicine

**DOI:** 10.3390/pharmaceutics10040170

**Published:** 2018-10-01

**Authors:** Jifen Zhang, Jiao Zhang, Shuai Wang, Tao Yi

**Affiliations:** 1 College of Pharmaceutical Sciences, Southwest University, Chongqing 400716, China; zhjf@swu.edu.cn (J.Z.); jiaozhang499@gmail.com (J.Z.); wangs53777@gmail.com (S.W.); 2 Neijiang Medical School of Sichuan Province, Neijiang 641100, China; 3 School of Health Sciences, Macao Polytechnic Institute, Macau 00853, China

**Keywords:** Pickering emulsion, puerarin, nanocrystals, oral bioavailability, poorly soluble drug, *Ligusticumchuanxiong* essential oil, volatile oil

## Abstract

In this study, an oral drug nanocrystals self-stabilized Pickering emulsion (NSSPE), which used nanocrystals of a poorly soluble ingredient from Puerariae Radix called puerarin as solid particle stabilizers and *Ligusticum* *chuanxiong* essential oil since the main oil phase had been developed to improve the oral bioavailability of puerarin. The appearance of emulsions, size and zeta potential of droplets, and content of puerarin in emulsified layer during a storage of six months at 4, 25, and 40 °C were investigated. The centrifugation stability at 4000× *g* was also studied. The micro-structure of emulsion droplets was characterized by a scanning electron micrograph (SEM), confocal laser scanning microscopy (CLSM), a fluorescence microscope (FM), and differential scanning calorimetry (DSC). The in vivo oral bioavailability of puerarin NSSPE was investigated in rats. Results showed that appearances of puerarin NSSPE kept stable after centrifugation at 4000× *g* for 15 min or storage for six months at 4, 25, and 40 °C. SEM, CLSM, FM, and DSC showed that the puerarin NSSPE had a stable core-shell structure of emulsion droplets formed by the adsorption of puerarin nanocrystals on the surface of oil droplets of mixed oil of *Ligusticum* *chuanxiong* essential oil and Labrafil M 1944 CS (9:1, *v*/*v*). The relative bioavailability of puerarin NSSPE to puerarin coarse powder suspension, nanocrystal suspension, and surfactant emulsion were 262.43%, 155.92%, and 223.65%, respectively. All these results indicated that puerarin nanocrystals could stabilize Pickering emulsion of *Ligusticum* *chuanxiong* essential oil without any other stabilizers and Pickering emulsion could improve the oral bioavailability of puerarin, which suggests that the drug nanocrystal self-stabilized Pickering emulsion as a promising oral drug delivery system for Traditional Chinese Medicine containing poorly soluble ingredients and volatile oils.

## 1. Introduction

Oral administration is the main and preferred route of administration for Traditional Chinese Medicine (TCM) regardless of the ancient or current methods because of its convenience, low cost, and high patient compliance compared with other routes. However, many of the bioactive ingredients of TCM were poorly soluble, which led to a low oral bioavailability and delivery problems. This decreased the efficacies of TCM or increased administration doses [1]. To address this natural shortcoming, many approaches have been developed including solubilization, inclusion compounds, solid dispersion, liposomes, nanoparticles, and micro-emulsion. However, within these techniques remained some shortcomings such as poor physical stability and a large amount of surfactants [2,3,4]. Moreover, they were usually used as a monomeric compound and were limited for TCM due to low drug loading.

Pickering emulsions are surfactant-free emulsions, which are stabilized by solid particles. The nearly irreversible adsorption of solid particles at the oil-water interface provides an effective steric barrier, which means Pickering emulsions have good physical stability especially high resistance to coalescence compared with surfactant-stabilized emulsion [5,6]. In addition, it is also eco-friendliness and low cost. Owing to these advantages, Pickering emulsions have attracted increasing research interests in recent two decades in pharmaceutical application fields for oral or topical delivery [7,8,9]. Considering that some pharmacologically active chemical compounds were water-insoluble, we have put forward an idea to develop a novel drug nanocrystals self-stabilized Pickering emulsion (NSSPE) in which a water-insoluble drug acted as a therapeutic agent as well as a stabilizing agent of emulsion droplets in a nanocrystal state. The idea had been proven to be available for silybin, which is a hydrophobic water-insoluble drug whose contact angle (θ) between air and water is 132 ± 5° and solubility was 51.06 ± 31.78 μg·mL^–1^. We had developed a Pickering emulsion stabilized by silybin nanocrystals with a droplet diameter of 27.3 ± 3.1 μm and verified that it could increase oral absorption of silybin four-fold when compared with silybin coarse powder [10]. Compared with traditional emulsions, NSSPE did not contain any surfactants or heterogeneous solid stabilizers, which resulted in greater safety and a higher drug loading capacity. As we all know, the wettability of particles is one key factor influencing the type and stability of Pickering emulsions [11,12]. Therefore, even if our previous study had proven NSSPE was a promising oral drug delivery system for hydrophobic water-insoluble drugs, whether the novel NSSPE could be suitable for poorly soluble but hydrophilic drugs is still uncertain.

Puerarin is a main active ingredient in Puerariae Radix, which is a traditional Chinese medicine herb and has protecting effects on the cardiovascular system, nervous system, osteoporosis, liver injury, and inflammation in vivo and in vitro [13]. It is regarded as a potential therapeutic agent to cerebrovascular diseases especially those caused by cerebral ischemia [14,15] because of its good activities of improving microcirculation, increasing blood flow in the brain, neuroprotection, and anti-platelet aggregation [13]. The main clinical route of puerarin at present was intravenous injection because its oral therapeutic effects were discounted by its poor water solubility and resultant low oral bioavailability of about 7% [16]. However, the frequent intravenous administration and a co-solvent of 1,2-propanediol in puerarin injection may lead to serious side effects such as allergy, hemolytic anemia, and drug fever [17]. To improve its oral bioavailability, many new formulations of puerarin such as nanocrystals [18,19,20], microemulsions [21], solid self-microemulsions [22], and solid lipid nanoparticles [23] have been studied. However, there was a large amount of surfactants in these pharmaceutical formulations, which might induce adverse effects such as allergy, tissue irritation, interactions with enterocytes, and even cell damage. This makes their use in biomedical applications a major concern [24,25]. Unlike silybin, puerarin was a hydrophilic drug with θ of 20 ± 5° between air and water. Its solubility was 65 times higher than that of silybin, which reached 3.28 ± 0.05 mg·mL^−1^. Therefore, it was of great value to explore whether puerarin could be made to be NSSPE for the application of NSSPE.

It is well-known that many volatile oils from TCM are pharmacologically active such as *Ligusticum chuanxiong* essential oil, which is one main biological active ingredient from *Ligusticum chuanxiong* Hort. It has been reported that *Ligusticum chuanxiong* essential oil had many pharmacological activities related to cerebrovascular diseases such as the improving function of blood vessels protecting nerve cells and sedation [26]. For volatile oils, emulsions were a favorite dosage form [27,28]. On the one hand, volatile oils could replace conventional oil excipients in emulsions such as semi-synthetic fatty acid glycerides, which markedly improves drug loading. On the other hand, their pharmacological activity could be synergistic with other ingredients, which creates a compound emulsion to improve the overall efficacy of TCM. Take *Ligusticum chuanxiong* essential oil for an example. It was synergically applied with puerarin in many Chinese medicine compounds such as Tongmai Formula and Naodesheng Formula, which were used to prevent and treat centrum cerebrovascular diseases clinically. *Ligusticum chuanxiong* essential oil was mainly composed of ligustilide, senkyunolide, *n*eocnidilide, *N*-butylphthalide, and 3-Butylidenephthalide, which accounted for about 80% and the content of fatty acid and esters was less than 10% [29,30]. This was completely different from other chemical fatty oils. Whether it could be used as a main oil phase of NSSPE to produce an oral compound Pickering emulsion also attracted our attention.

In this study, a new Pickering emulsion self-stabilized by hydrophilic puerarin nanocrystals (Pu-NSSPE) with *Ligusticum chuanxiong* essential oil when the main oil phase was prepared to improve the oral bioavailability of puerarin. The stability and microstructure of Pu-NSSPE were investigated. An in vivo study was performed on rats to compare the oral absorption of puerarin dosing in Pu-NSSPE with in coarse powder, nanocrystals, and traditional surfactant emulsions.

## 2. Materials and Methods

### 2.1. Chemicals and Reagents

Puerarin (purity > 98%) was purchased from Sichuan Yuxin Pharaceutical Co., Ltd. (Chengdu, China). Puerarin reference standard (purity > 98%) and parahydroxy benzaldehyde (internal standard, IS, purity > 98%) were purchased from Shanghai Yuanye Biotechnology Co., Ltd. (Shanghai, China). Labrafil M 1944 CS was purchased from GATTEFOSSé (Shanghai) Trading Co., Ltd. (Shanghai, China). *Ligusticum chuanxiong* essential oil (with a content of ligustilide 47.12%, Senkyunolide A 22.51%, Neocnidilide 4.19%, (Z,Z)-9,12-Octadecadienoic acid 4.13%, cis-9-Hexadecenal 3.5%, and 3-Butylidenephthalide 2.21%) was purchased from Jiangxi Xuesong Natural Medicinaliol Co., Ltd. (Ji’an, China). Nile red (purity > 98%) was purchased from Sigma (St. Louis, MO, USA) and Nile blue (purity > 75%) was purchased Adamas Co., Ltd. (Shanghai, China). HPLC-grade methanol was purchased from Sigma (St. Louis, MO, USA). Other solvents and chemicals were of an analytical grade.

### 2.2. Preparation and Characterization of Puerarin Nanocrystal Suspension (Pu-NCS)

Pu-NCS was prepared with a high pressure homogenization method. Puerarin coarse powder of 400 mg was dispersed in 80 mL pure water whose pH was pre-adjusted to 11.0 with 0.1 M NaOH using a high speed shearing machine (FA25, FLUKO fluid machinery manufacturing Co., Ltd., Shanghai, China) at 13,000 rpm for 2 min. The dispersion was then processed through a high pressure homogenizer (AH100D, ATS Engineering Ltd., suzhou, China) at 80 MPa for 3 min, which lasted about 15 cycles.

The mean particle size and zeta potential of Pu-NCS were measured using a Nanoseries ZS instrumen (Zetasizer Nano-ZS, Malvern Instruments, Malvern, UK). The morphology and size of nanocrystals were also checked by TEM. A drop of Pu-NCS was applied to a 200-mesh copper net and dried in the air. Then the shape and size of the nanocrystals were observed using TEM (FEI Talos F200S G2, Thermo Fisher Scientific Inc., Bleiswijk, the Netherlands). The physical stability of Pu-NCS was evaluated by observing the appearance of freshly prepared Pu-NCS during storage at room temperature.

The θ of particles was measured by the drop shape method [31]. A tablet was prepared by compressing lyophilized crystal at the pressure of 3 tons for 30 s. The tablet was immersed in a mixture of *Ligusticum chuanxiong* essential oil and Labrafil M 1944 CS (9:1, *v*/*v*) with a height of 1 cm for 30 min. Then, pure water of 2 μL was slowly dropped on the solid-oil interface with a micro-syringe equipped on a JC2000C contact angle measuring device (Shanghai Zhongchen digital technology equipment Co., Ltd., Shanghai, China). The shape of the water droplet was photographed after equilibrium and the data of θ were obtained using image analysis software.

### 2.3. Preparation of Pu-NSSPE

According to our previous study, mixed oil of *Ligusticum chuanxiong* essential oil and Labrafil M 1944 CS (9:1, *v*/*v*) was used as the oil phase of Pu-NSSPE [32]. In addition, 63 mL of fresh Pu-NCS obtained above was mixed with 7 mL of the oil phase by a high speed shearing machine at 13,000 rpm for 2 min and then processed again through a high pressure homogenizer at 80 MPa for 3 min.

### 2.4. Stability of Pu-NSSPE

The centrifugation stability of Pu-NSSPE was studied first. The freshly prepared emulsions were centrifuged at 4000 g for 15 min to observe whether there was sedimentation, creaming, or coalescence. At the same time, the turbidity of emulsion was monitored at 500 nm by a U-3010 UV-Vis spectrophotometer (Hitachi, Tokyo, Japan) after emulsion was diluted 200-fold with pure water. The centrifugation stability (CS) was assessed below [33].

CS = *A*_t_/*A*_0_ × 100%

where *A*_0_ and *A*_t_ were the absorbance at 500 nm before and after centrifugation, respectively.

In addition, the storage stabilities of Pu-NSSPE were investigated. The fresh Pu-NSSPE was transferred into two glass vessels and stored at 4, 25, and 40 °C, respectively. The emulsions were studied at 0, 1, 2, 3, 4, 5, and 6 months, respectively. At each time point, the appearances were observed and stability indexes (SI) were calculated below.

SI = *H*_t_/*H*_0_

where *H*_t_ was the height of the emulsion layer at a certain time and *H*_0_ was the total height of the samples [34].

The volume mean diameter (*d*_4,3_) of emulsion droplets was calculated based on the optical microscopy of NSSPE captured by an optical microscope equipped with a Cool SNAP Photomerics optical acquisition system (Chongqing Optec Instrument Co., Ltd., Chongqing, China) [35] by considering 200 droplets for each emulsion. The calculation equation is shown below.

$$d_{4,3}=\frac{\Sigma {\mathrm{di}}^{4}}{\Sigma {\mathrm{di}}^{3}}$$

in which *di* was the diameter of one droplet.

The zeta potential of the oil droplets was determined using a Zetasizer Nano-ZS instrument (Malvern Instruments, Worcestershire, UK) after the emulsion was diluted 100-fold with pure water. Emulsions of 20 μL were dissolved in a 3 mL mixture of methanol and chloroform (1:2) and then diluted to 10 mL with methanol. The contents of puerarin were determined by a HPLC method. An Ultimate^®^ XB-C18 column (250 mm × 4.6 mm, 5µm) was used. The mobile phase was methanol and 0.1% citric acid (29:71). The flow rate was 0.6 mL·min^−1^ and the detection wavelength was 250 nm.

### 2.5. Microstructure Characterization of Pu-NSSPE

#### 2.5.1. Scanning Electron Microscopy

One drop of Pu-NSSPE was spread onto a clean thin glass and air-dried at room temperature. The morphology was observed by SEM (JSM-6510LV, JEOL Ltd., Tokyo, Japan) after being vacuum-coated with a gold-palladium film. At the same time, a blank emulsion prepared using the same procedure as Pu-NSSPE without puerarin was also observed as a control.

#### 2.5.2. Fluorescence Microscope

To justify the adsorption behavior of nanocrystals at the oil-water interface, Pu-NSSPE was observed by FM directly because puerarin had a strong fluorescence when excited by UV-lights [36]. A drop of emulsion was placed on a glass surface and visualized with a fluorescence inverted microscope (DFC310 FX, Leica, Solms, Germany) immediately.

#### 2.5.3. Confocal Laser Scanning Microscope

For CLSM observation, labeled emulsions were prepared as follows. Nile blue (0.1%) was first added to the water phase and Nile red (0.1%) was dissolved in the oil phase, respectively. The other preparation procedures were the same as Pu-NSSPE. The labeled emulsion was observed by a Nikon N-STORM CLSM (Nikon, Tokyo, Japan). The fluorescent dyes were excited at 488 nm for Nile red and at 633 nm for Nile blue.

#### 2.5.4. Differential Scanning Calorimetry

DSC was performed with DSC 200PC (Netzsch Ltd., Selb, Germany). Pu-NCS was lyophilized (CoolSafe 110-4 Pro, ScanLaf Ltd., Lynge, Denmark) without any additives to obtain a dry sample. The puerarin nanocrystals adsorbed onto the surface of oil droplets in Pu-NSSPE were collected and dried at room temperature after Pu-NSSPE was centrifuged at 183, 960× *g* for 1 h at 4 °C. Three samples including puerarin course powder, lyophilized Pu-NCS powder, and adsorbed puerarin nanocrystals in Pu-NSSPE were analyzed. The thermal behavior was analyzed from 40 °C to 250 °C by using a heating rate of 10 °C/min with approximately 5 mg of each sample sealed in an aluminum pan, respectively.

### 2.6. Pharmacokinetic Study in Rats

#### 2.6.1. Preparation of Control Samples

Pu-NCS, puerarin course powder suspension (Pu-CPS), and puerarin surfactant emulsion (Pu-SE) were also used as controls. To obtain Pu-CPS, puerarin course powder of 50 mg was added into 10 mL pure water (pH was pre-adjusted to 11 with 0.1 M NaOH) and vortexed for 2 min. Pu-SE was prepared as follows. Mixed oil phase of 7 mL, puerarin coarse powder of 315 mg, and tween 80 of 700 μL were mixed with 63 mL of pure water (pH was pre-adjusted to 11.0 with 0.1 M NaOH) by a high speed shearing machine at 13,000 rpm for 3 min and then processed through a high pressure homogenizer at 80 MPa for 3 min.

#### 2.6.2. Drug Administration and Sampling

Sprague-Dawley rats (180 to 200 g, half male and half female) of clean grade were purchased from the Chongqing Academy of Chinese Materia Medica. The rats were housed in an SPF animal laboratory (22 to 25 °C, 50% to 60% relative humidity, and a 12 h cycle of light and dark) of Southwest University with unlimited access to food and water. All animal experiments were performed in accordance with China’s Guidelines for Care and Use of Laboratory Animals and were approved by the Animal Ethics Committee of Southwest University (Chongqing, China) (No. 2016-09).

A total of 24 rats were divided randomly into 4 groups after an overnight fast. Pu-CPS, Pu-NCS, Pu-SE, and Pu-NSSPE were intra-gastrically administered to each group at a dose of 100 mg·kg^–1^ (equivalent to puerarin), respectively. Blood samples were collected at 0.083, 0.16, 0.25, 0.5, 1, 2, 3, 4, 6, 8, and 12 h after administration and placed into pre-heparinized centrifuge tubes immediately. Blood samples were centrifuged at 3500 rpm for 15 min and stored at −20 °C until analysis.

#### 2.6.3. Puerarin Blood Concentration Analysis

For analysis, 10 μL of parahydroxy benzaldehyde as IS solution (6040 ng/mL), 260 μL methanol, and 750 μL acetonitrile were added to a 200 μL plasma sample and vortex-mixed for 2 min. The mixtures were centrifuged at 15,000 rpm for 10 min. The supernatants were vacuum-dried at 40 °C. The residues left were reconstituted in 200 μL methanol and centrifuged at 15,000 rpm for 10 min. The supernatants were analyzed by HPLC (LC-20A HPLC system, Shimadzu Corporation, Kyoto, Japan). An Ultimate XB–C18 column (Waters, 5 μm, 4.6 mm × 250 mm) was used. The mobile phase consisted of methanol and 0.1% formic acid (25:75, *v*/*v*). The flow rate was 1.0 mL/min and the detection wavelength was 250 nm.

### 2.7. Data and Statistical Analyses

The main pharmacokinetic parameters of puerarin, i.e. the area under the plasma concentration-time curve (AUC), elimination half-life (*t*_1/2_), and mean residence time (MRT) were calculated by using non-compartmental analysis by the DAS 2.0 software package (Mathematical Pharmacology Professional Committee of China, Shanghai, China). The maximal plasma concentration (*C*_max_) and the time to reach *C*_max_ (*T*_max_) were directly obtained from the Plasma concentration–time curve. All data were expressed as mean ± standard deviation (S.D.). One-way ANOVA was used to test the differences between groups and *P* < 0.05 or *P* < 0.01 was considered to be a significant difference.

## 3. Results and Discussions

### 3.1. Characterization of Pu-NCS

**Figure 1:**
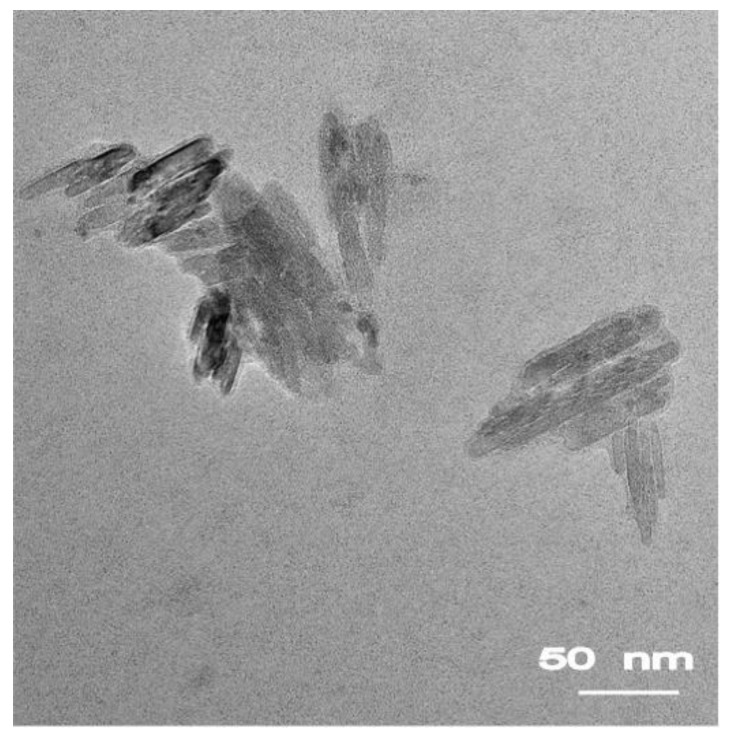
TEM images of puerarin nanocrystals in puerarin nanocrystal suspension (Pu-NCS).

The particle size of puerarin nanocrystals was 390.9 ± 78.5 nm and the zeta potential was −44.7 ± 3.0 mV. Figure 1 showed that puerarin nanocrystals in Pu-NCS were slender columns with sizes of about 50 to 100 nm. Obvious flocculation was observed within 1 h when Pu-NCS was stored at room temperature and the resultant loose aggregates could not be re-dispersed, which shows that the physical stability of Pu-NCS was poor.

The θ of puerarin nanocrystals between water and mixed oil was composed of *Ligusticum chuanxiong* essential oil and Labrafil M 1944 CS (9:1, *v*/*v*) was 81 ± 4°. As Pickering emulsion stabilizers, solid particles should have a proper partial wettability. The optimal stability of Pickering emulsions could be ensured when the θ of particle stabilizer is close to 90° [11,37,38]. Therefore, hydrophilic puerarin nanocrystals should be liable to stabilize O/W emulsions with *Ligusticum chuanxiong* essential oil and Labrafil M 1944 CS (9:1, *v*/*v*) as the oil phase theoretically.

In our previous study, θ of hydrophobic silybin between water and oil (Capmul^®^ MCM C8) was similar to that between water and air (123 ± 2° and 132 ± 5°, respectively [10]). However, for hydrophilic puerarin, θ between water and mixed oil (*Ligusticum chuanxiong* essential oil and Labrafil M 1944 CS, 9:1, *v*/*v*) was much larger than that between water and air (20 ± 5°). We also measured θ of puerarin between water and Capmul C8, Labrafil M 1944 CS, isopropyl myristate, *Ligusticum chuanxiong* essential oil, and soybean oil, which were 49 ± 5°, 14 ± 1°, 32 ± 2°, 70 ± 2°, and 25 ± 4°, respectively [32]. It suggested that the oil phase may have great influence on wettability of hydrophilic puerarin and a stable Pickering emulsion could be prepared with hydrophilic puerarin nanocrystals as solid particles by selecting a proper oil phase.

### 3.2. Stability of Pu-NSSPE

**Figure 2:**
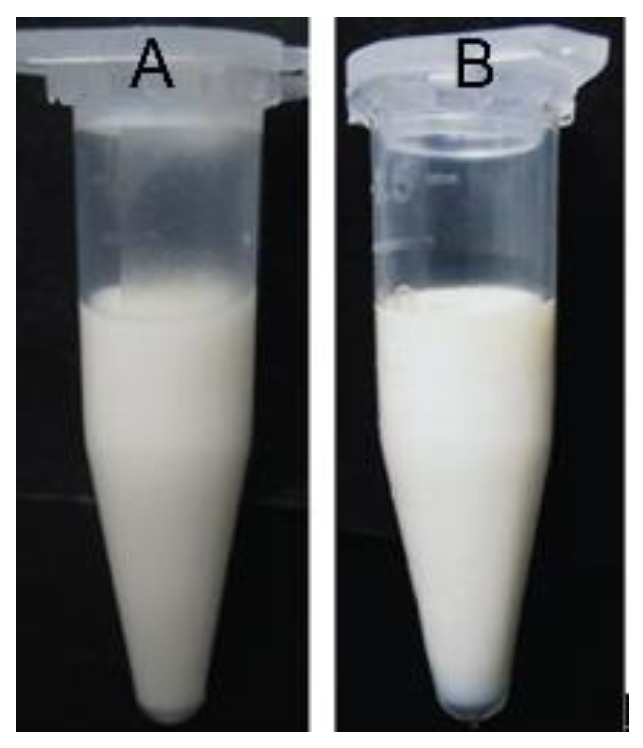
Appearances of puerarin nanocrystals self-stabilized Pickering emulsion (Pu-NSSPE) (**A**) before and (**B**) after centrifugation at 4000× *g* for 15 min.

No sedimentation, creaming, or coalescence was observed after centrifugation of Pu-NSSPE at 4000× *g* for 15 min (Figure 2). The absorbance of emulsified samples were diluted 200-fold at 500 nm before and after centrifugation were 0.702 ± 0.003 and 0.692 ± 0.004, respectively, and there was no obvious difference (*P* > 0.05). CS was as high as 0.986, which indicated that Pu-NSSPE had a good centrifugation stability.

**Figure 3:**
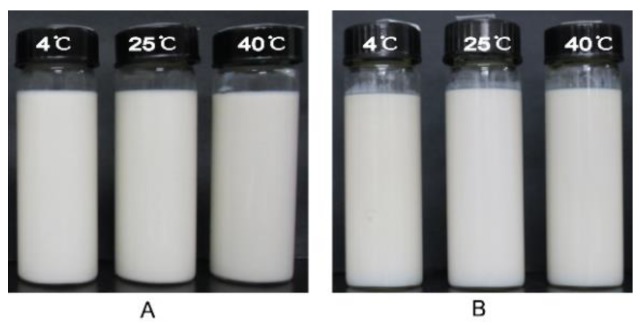
Photographs of Pu-NSSPE (**A**) freshly prepared and (**B**) stored at 4, 25, and 40 °C for 6 months.

**Figure 4:**
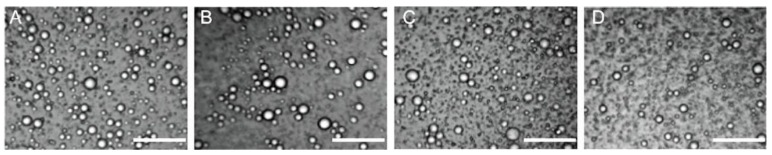
Optical micrographs of Pu-NSSPE (**A**) freshly prepared and stored at (**B**) 4, (**C**) 25, and (**D**) 40 °C for 6 months. Bar 100μm.

**Figure 5:**
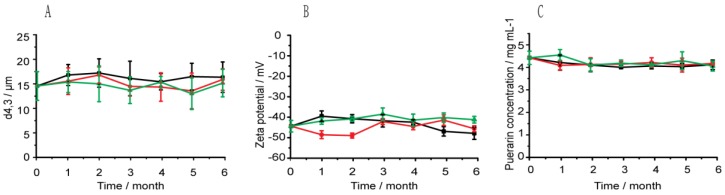
The (**A**) Droplet sizes, (**B**) zeta potentials, and (**C**) puerarin concentrations of Pu-NSSPE during 6 months storage at 4 (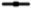), 25 (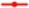), and 40 (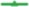) °C.

As shown in Figure 3, there were no obvious changes for appearance of Pu-NSSPE after storage for 6 months at 4, 25, and 40 °C either without any sedimentation, creaming, or coalescence observed. The SIs for all samples were 1. This was significantly different from Pu-NCS, which subsided obviously within 1 h after preparation. The optical micrographs of emulsion droplets were shown in Figure 4 and the droplet sizes, zeta potentials, and drug contents were shown in Figure 5. All droplets of Pu-NSSPE were spherical. The *d*_4,3_ of droplets was 12.4 ± 2.9 μm for fresh Pu-NSSPE, which did not change significantly after 6 months of storage with 14.2 ± 3.1, 13.7 ± 2.2, and 13.0 ± 2.8 μm at 4, 25, and 40 °C, respectively. Though puerarin concentration during storage fluctuated slightly between 4.19 and 4.58 mg/mL, there were no significant differences by using One-way ANOVA analysis (*P* > 0.1). Zeta potentials of oil droplets were also stable during storage. The initial zeta potential was −44.7 ± 2.2 mV and the final zeta potentials after a storage of 6 months at 4, 25, and 40 °C were −47.4 ± 2.3, −45.7 ± 1.1, and −42.3 ± 1.3 mV, respectively. All these results showed Pu-NSSPE had a good stability within 6 months even if stored at high (40 °C) and low (4 °C) temperatures.

### 3.3. Microstructure Characterization of Pu-NSSPE

**Figure 6:**
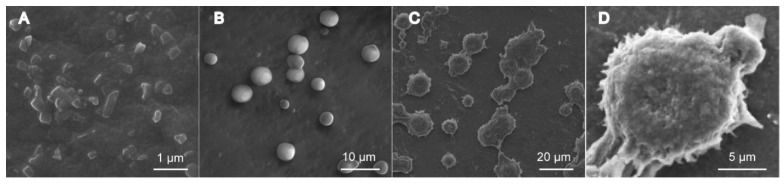
SEM images of (**A**) puerarin nanocrystals, (**B**) blank oil droplet, (**C**) Pu-NSSPE, and (**D**) emulsion droplet surface of Pu-NSSPE.

Figure 6 showed that the droplet surface of Pu-NSSPE was not as smooth as blank emulsion, which proved that Pu-NSSPE may have a different surface characterization from blank emulsion. A severe adhesion of emulsion droplets was observed in Pu-NSSPE. This may be due to solvent evaporation during the preparation of samples for SEM. To characterize the surface adsorption of puerarin nanocrystals, other experiments should be conducted further such as CLSM or cryo-SEM.

**Figure 7:**
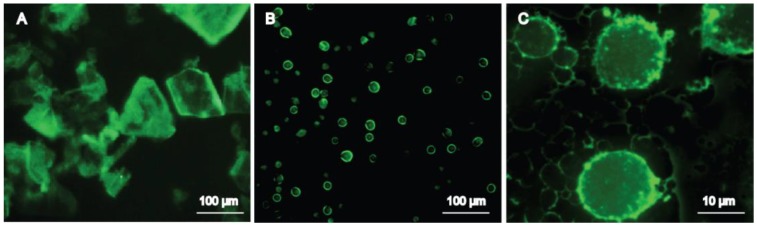
Fluorescence micrographics of (**A**) puerarin crude material, (**B**,**C**) Pu-NSSPE.

**Figure 8:**
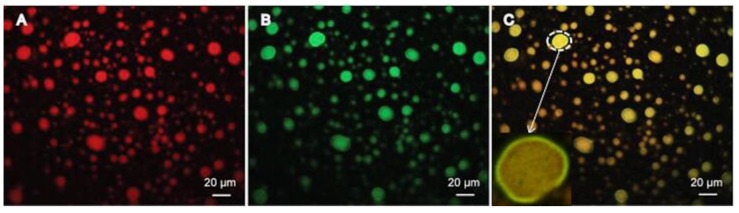
CLSM images of Pu-NSSPE (**A**) excited at 488 nm for Nile red, (**B**) excited at 633 nm for Nile blue, and (**C**) the combined image of A and B.

Strong fluorescence was observed by FM for puerarin raw material (Figure 7A). In Figure 7B,C, strong fluorescence was observed on the surface of emulsion droplets, which proves that puerarin nanocrystals were adsorbed on the surface of oil droplets. However, the fluorescence in the interior of emulsion droplets was much weaker, which was due to the small solubility of puerarin in oil phase. Staining the oil phase by Nile red and puerarin nanocrystals by Nile blue, respectively, microstructures of Pu-NSSPE were also studied with CLSM. As shown in Figure 8A, emulsion droplets were all red with Nile red distributed in the oil uniformly. In Figure 8B, strong green fluorescence of Nile blue around emulsion droplets could be observed, which indicated that substances around droplets were different from those inside droplets. Figure 8C was a combined image of A and B. Emulsion droplets showed yellow due to an overlay of green and red color. Green fluorescence was still observed around yellow droplets, which illustrated that the oil droplets were wrapped up in the green Nile blue. This indicated a stable oil (core) and puerarin nanocrystals (shell) structure.

**Figure 9:**
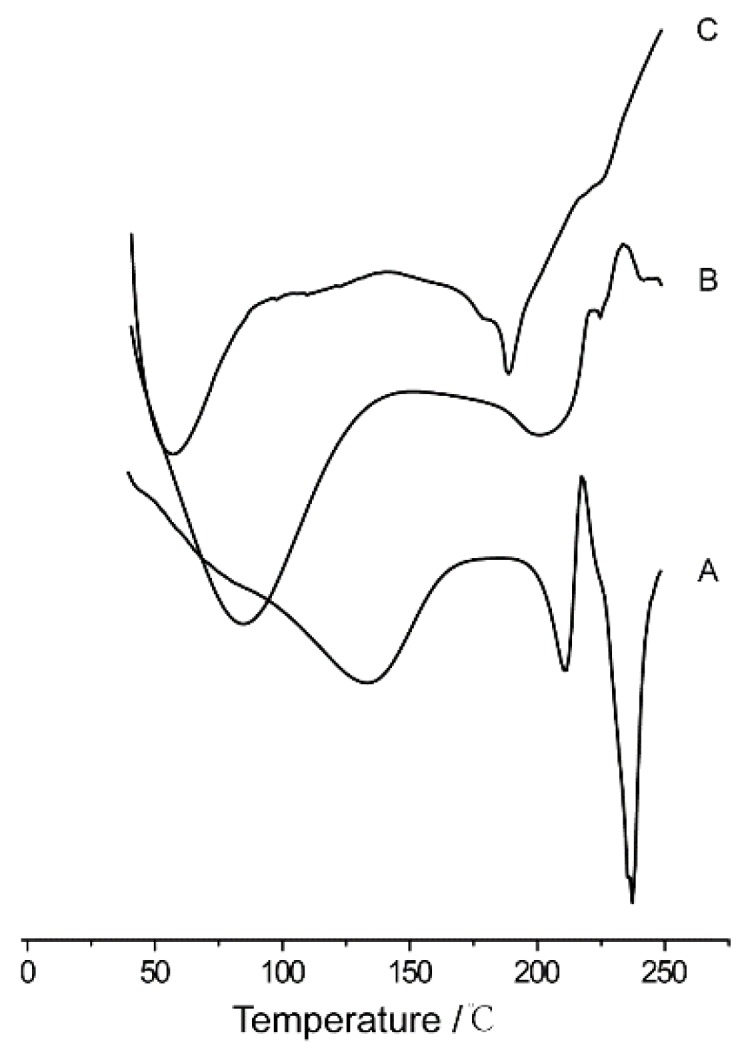
DSC curves of (**A**) puerarin crude material, (**B**) lyophilized Pu-NCS, and (**C**) adsorbed puerarin in Pu-NSSPE.

DSC also verified that there were puerarin nanocrystals in Pu-NSSPE. However, there were some differences among the DSC curves of puerarin crude material (A), lyophilized Pu-NCS (B), and adsorbed puerarin in Pu-NSSPE (C). As shown in Figure 9, the puerarin coarse powder exhibited two sharp endothermic peaks at 210 °C and 238 °C, which was in accord with the document [18]. The melting points of lyophilized Pu-NCS were slightly changed to 202 °C and 225 °C, respectively. The shapes of peaks were also changed with a wider peak at 202 °C and a weaker peak at 225 °C. By contrast, the endothermic peak of adsorbed puerarin in Pu-NSSPE moved from 202 °C to 189 °C and the other small peak at 225 °C disappeared. The reasons may be that the interaction of puerarin nanocrystals with mixed oil under high pressure homogenization caused a partial change of puerarin nanocrystals because of the crystal polymorphism of puerarin [19]. It also may be because that part of nanocrystals re-dissolved. Overall, nanocrystals of puerarin still existed whether it was recrystallized or undissolved.

Results from SEM, FA, CLSM, and DSC all supported the conclusion that puerarin nanocrystals were adsorbed onto the surface of oil droplets in Pu-NSSPE. It was reported that when 30° < θ < 150°, the energy required for detaching the particles from the oil-water interface would be several orders larger than the kinetic energy for Brownian motion [39]. Therefore, θ of 81 ± 4° could make the adsorption of puerarin nanocrystals at the oil-water interface irreversible. It was the irreversible adsorption that led to a high stability of Pu-NSSPE.

### 3.4. Pharmacokinetic Study in Rats

Four different formulations known as Pu-CPS, Pu-NCS, Pu-SE, and Pu-NSSPE were i.g. administered to rats. The puerarin content for Pu-CPS, Pu-NCS, Pu-SE, and Pu-NSSPE were 4.55 ± 0.25, 4.46 ± 0.28, 4.41 ± 0.22, and 4.49 ± 0.21 mg/mL, respectively. The size of particles of Pu-CPS and Pu-NCS or emulsion droplets of Pu-SE and Pu-NSSPE were 91.3 ± 1.7 μm, 363.5 ± 61.5 nm, 3.8 ± 0.7 μm, and 14.4 ± 2.9 μm, respectively. The final pH of Pu-CPS, Pu-NCS, Pu-SE, and Pu-NSSPE were 6.7 ± 0.1, 6.9 ± 0.1, 6.2 ± 0.1, and 6.1 ± 0.1, respectively.

**Figure 10:**
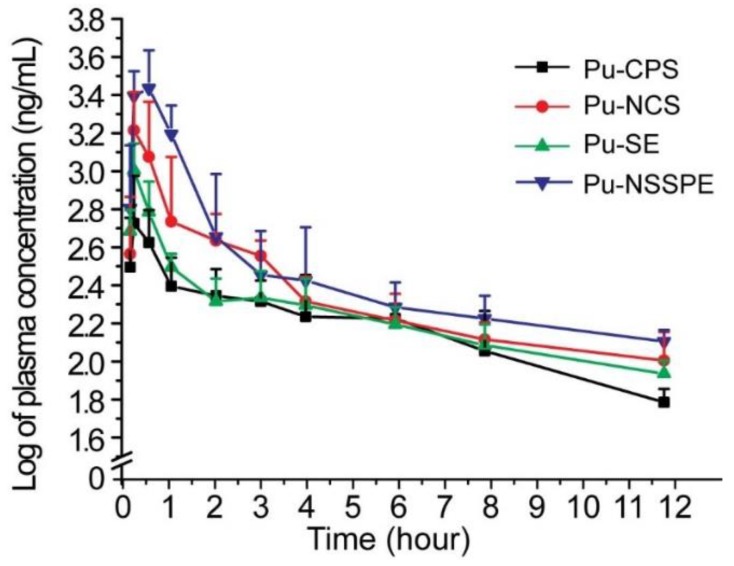
The log diagram of plasma concentration–time curves of Pu-CPS, Pu-NCS, Pu-SE, and Pu-NSSPE after a single administration at the puerarin dose of 200 mg·kg**^–^**^1^ in rats (*n* = 6, $\bar{x}$ ± SD).

Plasma concentration–time curves were presented in Figure 10 and the main pharmacokinetic parameters were listed in Table 1. There were obvious differences among the four formulations within 4 h after administration and Pu-NSSPE showed outstanding features. As shown in Table 1, *C*_max_ of Pu-NSSPE was 4.09 times, 5.16 times, and 0.71 times higher than that of Pu-CPS, Pu-NCS, and Pu-SE, respectively (*P* < 0.01). T_1/2_ for Pu-CPS was much lower than those for the other three formulations. However, there were no significant differences in T_1/2_ among Pu-NCS, Pu-SE, and Pu-NSSPE. As far as AUC_0-t_ was concerned, there was no obvious difference between Pu-CPS and Pu-SE, but Pu-NCS and Pu-NSSPE both showed much higher AUC_0-t_ than the other two formulations. The relative bioavailability of Pu-NSSPE to Pu-CPS, Pu-NCS, and Pu-SE were 262.43%, 155.92%, and 223.65%, respectively. It could be concluded that NSSPE could significantly improve the oral bioavailability of puerarin.

Puerarin was categorized as the IV drug of the BCS because of its low solubility and low intestinal permeability values [40], which results in a low AUC of Pu-CPS. The adsorption of Pu-NSSPE in vivo was dramatically higher than that of Pu-CPS, which had nothing to do with *Ligusticum chuanxiong* essential oil because AUC of Pu-SE with the same oil was not significantly different from that of Pu-CPS. Other studies also showed that Pickering emulsions that are not ordinary emulsions could increase the adsorption of poorly soluble drugs [41,42]. Pu-NSSPE increased the AUC and *C*_max_ in comparison to Pu-SE even though they were both O/W emulsion and the latter had relatively smaller droplet sizes. It suggested that differences in the size of initial emulsion droplets did not have a pronounced effect on the extent of puerarin absorption. In addition, Pu-NCS had a higher AUC than Pu-CPS. Therefore, it could be inferred that the highest adsorption of puerarin in Pu-NSSPE should be relative to the nanocrystal state of puerarin. Pu-NSSPE had a higher AUC than Pu-NCS, which could be due to the partial change in crystal form of puerarin in Pu-NSSPE as proven by DSC.

## 4. Conclusions

In the study, a new compound Pickering emulsion, Pu-NSSPE, was developed with puerarin nanocrystals used as a solid particle stabilizer for Pickering emulsion and *Ligusticum chuanxiong* essential oil as the main oil phase. In this compound Pickering emulsion, puerarin and *Ligusticum chuanxiong* essential oil both acted as therapeutic substances as well as adjuvants of emulsion. Compared with other conventional emulsions, there were no other surfactants or polymer stabilizers and the amount of chemical oil was reduced. With a core–shell structure formed by adsorbing puerarin nanocrystals on the surface of oil droplets and resulting in a good physical stability, Pu-NSSPE could increase the oral bioavailability of puerarin significantly. It could be concluded that NSSPE may be a promising oral drug delivery system especially for TCM with poorly water soluble drugs and volatile oils.

## Figures and Tables

**Table 1:** Pharmacokinetic parameters of Pu-CPS, Pu-NCS, Pu-SE, and Pu-NSSPE after a single administration at the puerarin dose of 200 mg/kg in rats. *n* = 6, $\bar{x}$ ± *s*.

Parameter	Pu-CPS	Pu-NCS	Pu-SE	Pu-NSSPE
AUC_0–*t*_/ng·mL**^–1^**·h	1903.05 ± 272.12	3203.11 ± 1021.48 ^#^	2233.01 ± 341.56	4994.22 ± 1650.83 ^# #,Δ,**^
*T*_max_/h	0.22 ± 0.14	0.22 ± 0.14	0.17 ± 0.00	0.33 ± 0.18 *
*C*_max_/ng·mL**^–1^**	634.17 ± 292.77	1881.44 ± 731.84 ^# #^	1021.38 ± 359.49 ^Δ^	3226.14 ± 726.64 ^# #,ΔΔ,^**
*t*_1/2_/h	3.14 ± 1.20	5.48 ± 1.89 ^#^	5.32 ± 1.56 ^# #^	5.57 ± 1.16 ^# #^
MRT/h	3.82 ± 0.98	3.16 ± 1.10 ^# #^	3.86 ± 0.60	2.09 ± 0.60 ^# #,Δ,^**

^#^*p* < 0.05, ^##^ *p* < 0.01 vs. the Pu-CPS group. ^Δ^ *p* < 0.05, ^ΔΔ^ *p* < 0.01 vs. the Pu-NCS group. * *p* < 0.05, ** *p* < 0.01 vs. the Pu-SE group.
